# Single-cell profiling reveals transcriptome dynamics during bovine oocyte growth

**DOI:** 10.1186/s12864-024-10234-0

**Published:** 2024-04-06

**Authors:** Lais Barbosa Latorraca, António Galvão, Maria Belen Rabaglino, Julieta Maria D’Augero, Gavin Kelsey, Trudee Fair

**Affiliations:** 1 School of Agriculture and Food Science, University College Dublin, Dublin, Ireland; 2 Epigenetics Programme, The Babraham Institute, Cambridge, UK; 3 Centre for Trophoblast Research, University of Cambridge, Cambridge, UK; 4 Wellcome-MRC Institute of Metabolic Science-Metabolic Research Laboratories, Cambridge, UK; 5 Department of Comparative Biomedical Sciences, Royal Veterinary College, London, UK; 6 Department of Population Health Sciences, Faculty of Veterinary Medicine, Utrecht University, Yalelaan 7, 3584 CL Utrecht, The Netherlands

**Keywords:** Oogenesis, RNA sequencing, Gene Expression, Cattle

## Abstract

**Background:**

Mammalian follicle development is characterized by extensive changes in morphology, endocrine responsiveness, and function, providing the optimum environment for oocyte growth, development, and resumption of meiosis. In cattle, the first signs of transcription activation in the oocyte are observed in the secondary follicle, later than during mouse and human oogenesis. While many studies have generated extensive datasets characterizing gene expression in bovine oocytes, they are mostly limited to the analysis of fully grown and matured oocytes. The aim of the present study was to apply single-cell RNA sequencing to interrogate the transcriptome of the growing bovine oocyte from the secondary follicle stage through to the mid-antral follicle stage.

**Results:**

Single-cell RNA-seq libraries were generated from oocytes of known diameters (< 60 to > 120 μm), and datasets were binned into non-overlapping size groups for downstream analysis. Combining the results of weighted gene co-expression network and Trendy analyses, and differently expressed genes (DEGs) between size groups, we identified a decrease in oxidative phosphorylation and an increase in maternal -genes and transcription regulators across the bovine oocyte growth phase. In addition, around 5,000 genes did not change in expression, revealing a cohort of stable genes. An interesting switch in gene expression profile was noted in oocytes greater than 100 μm in diameter, when the expression of genes related to cytoplasmic activities was replaced by genes related to nuclear activities (e.g., chromosome segregation). The highest number of DEGs were detected in the comparison of oocytes 100–109 versus 110–119 μm in diameter, revealing a profound change in the molecular profile of oocytes at the end of their growth phase.

**Conclusions:**

The current study provides a unique dataset of the key genes and pathways characteristic of each stage of oocyte development, contributing an important resource for a greater understanding of bovine oogenesis.

**Supplementary Information:**

The online version contains supplementary material available at 10.1186/s12864-024-10234-0.

## Background

The application of RNA-sequencing (RNA-seq) analysis to mouse oocyte biology research has provided novel information on the transcripts, their co-factors and regulators which drive the development of the female germline from the formation of the primordial germ cells through differentiation to oogonia, and progression through oogenesis [1–4]. The duration of this latter stage, i.e., oocyte growth from activation of primordial follicles until the pre-ovulatory follicle stage, takes 10 to 12 days in mice [5], but more than 120 days in humans [6, 7] and cattle [8]. During this period, dynamic morphological changes take place in the follicular cells with modifications in number, morphology, metabolic activity, cell differentiation, and responsiveness to hormones [9, 10]. Similarly, once activated to grow, the oocyte embarks on a phase of dramatic molecular and morphological transformation, involving a greater than fivefold increase in volume, synthesis and reorganization of nuclear and cytoplasmic organelles [11–13], and the establishment of the oocyte epigenome and transcriptome [1, 2, 14], all of which must sustain oocyte resumption and completion of meiotic maturation, fertilization, early embryonic cleavage divisions, and activation of the embryonic genome [15].

The employment of high-throughput multi-omic technologies to interrogate the contribution of both candidate and unknown factors to the above-mentioned events has centred on the mouse model. The first deep RNA-seq analysis of non-growing and growing murine oocytes was published in 2015 [1], which was succeeded by an analysis of murine oocytes ranging from 10 to 65 μm in diameter [14]. Together, these studies showed that the transcriptional programme of the murine oocyte is largely set up in oocytes ranging from 10 to 40 μm in diameter, likely from primordial follicles and that subsequent changes to the oocyte transcriptome are relatively modest during murine oocyte growth. A comparable analysis of human oocytes and granulosa cells across the follicle developmental trajectory from the primordial to pre-ovulatory stage suggested a more dynamic transcriptome in humans compared to murine oocytes, with the presence of higher numbers of differently expressed genes (DEGs) in the comparisons of oocytes from different stages of follicle development [16]. However, comparable information for oocytes from other mammalian species is limited.

The first study investigating the onset of RNA transcription in bovine oocytes employed ^3^uridine incorporation coupled with autoradiography and identified the secondary follicle stage [12]. Later, using real-time PCR analysis of candidate genes, Bessa et al. (2013) [17] reported a low level of mRNA expression for many assayed genes in oocytes from primordial up to small secondary follicles (25 to 60 μm in diameter), with a significant increase only in oocytes from large secondary follicles (65 to 85 μm in diameter). Such pronounced differences in the transcription dynamics in murine, human, and bovine oocytes likely reflect the duration and kinetics of the oocyte growth phase in these species and highlight the potential for some aspects of the molecular regulation of mammalian oocyte growth and acquisition of developmental competence to be species-specific.

In contrast to the vast repertoire of data from mouse, most RNA-seq analyses of bovine oocytes have been carried out at fully-grown germinal vesicle (GV) or metaphase II (MII) -stages, where the emphasis has been on identifying the impact of different in vivo or in vitro environments [18, 19], vitrification [20], heat stress [21, 22], ovarian hormonal manipulation [23, 24], and in vitro culture systems [25], on the oocyte transcriptome. The evolution of the oocyte transcriptome as it progresses through and completes the growth phase has not been described to date. Such a dataset would provide a unique reference resource for understanding the molecular basis for oocyte acquisition of developmental competence in cattle. Therefore, the present study aimed to redress this knowledge gap by carrying out single-cell RNA-seq (scRNA-seq) on bovine oocytes from the secondary follicle up to the mid-antral follicle -stage of development. The objective of the current study was to generate a high-quality transcriptome annotation of bovine oocytes which captures the dynamic changes in gene expression during the oocyte growth phase and identifies hallmark genes and pathways associated with key stages of oocyte development. Such knowledge will inform the development of appropriate in vitro systems for bovine oocyte growth which attend to the specific needs of each phase of development.

## Material and methods

### Oocyte collection

Bovine (*Bos taurus*) ovaries were collected at a local abattoir and returned to the laboratory in cold PBS. Fully-grown oocytes were removed first by aspirating 3–8 mm surface visible follicles. Subsequently, the ovaries were butterflied open, submerged in cold Dissection medium (TCM199 supplemented with 0.4% BSA fraction V, 0.164 mM penicillin, 0.048 mM streptomycin, 1,790 units/L heparin, and 5 μM cilostamide [26]), and growing oocytes from late secondary to early antral follicles were liberated by slicing the ovarian cortex. Subsequently, the medium containing the oocytes was filtered through a descending series of mesh filters, including 260 μm mesh, 100 and 40 μm PluriStrainer® filters. Recovered oocytes were washed in cold Dissection medium, denuded by pipetting, and allocated singly to drops of cold PBS, where their diameter was measured under a Nikon Eclipse TE2000-S microscope and NIS Elements BR 5.02.00 64-bit software. Measured oocytes were snap-frozen in 4 μL PBS + 4 μL RTL-Plus buffer (Qiagen) in 0.2 mL PCR microtubes.

### Single-cell RNA sequencing

RNA from individual oocytes was isolated using the Genome & Transcriptome protocol [27], and cDNA conversion, amplification, and purification were performed as described by [28]. Briefly, oocyte lysates were transferred to a 96-well plate and incubated with Dynabeads (MyOne Streptavidin C1, Life Technologies) annealed to Smart-seq2 oligo-dTs [29, 30] to capture polyadenylated mRNA. The remaining lysate containing the DNA was transferred to a new 96 well-plate for a subsequent bisulphite conversion analysis. The beads containing the mRNA were diluted in reverse transcription master mix using SuperScript™ II Reverse Transcriptase (Invitrogen). cDNA was amplified (14 cycles) using KAPA HiFi HotStart ReadyMix (Roche), purified with Ampure XP beads with a 1:0.9 ratio, and eluted into 20 μL of water [29, 30]. Amplified cDNA was quantified using a High Sensitivity DNA Assay chip (Agilent Technologies), and libraries were prepared using the Nextera XT Kit (Illumina) with ~ 300 pg of cDNA in two different replicates (Supplementary Fig. S1). The individual transcriptome of 179 oocytes was sequenced on the NextSeq500 HighOutput 150 bp Single End (replicate 1) and 75 bp Single End (replicate 2) with a sequence depth of ~ 2 million reads.

### Data processing and quality control

Trim Galore (version 0.6.6) was used to trim raw sequence reads by removing adapter contamination and poor-quality reads with less than 20 Phred score. Only reads with a minimum 20 bp sequence length after trimming were retained for further processing [31, 32]. The data was mapped to the bovine reference genome (bosTau 9) and generated genome index with ARS-UCD1.2 assembly using HISAT2. A total of 632,700,000 reads were sequenced (from 500,000 to 12,200,000 reads/sample) with 86% being uniquely mapped to the bovine genome (from 384,760 to 10,495,372 reads/sample) (Supplementary Table S1). A total of 21,218 transcripts were identified using SeqMonk software (version 11.0.10; Babraham Institute; https://www.bioinformatics.babraham.ac.uk/projects/seqmonk/). Raw counts were quantified with the SeqMonk RNA-seq quantification pipeline.

Sample quality control was performed using RStudio software (2023.06.0 + 421) as follows: Genes presenting zero read counts in all samples were designated ‘not expressed’ and removed from the dataset (3,221 genes). Samples with < 100,000 reads and < 2,500 expressed genes (genes with more than 1 count) were also removed. Finally, samples contaminated with noncoding mitochondrial genes (i.e., more than 3% of reads mapped to mitochondrial genes) were also excluded from further analysis. A final sample set of 165 oocytes and a dataset of 16,979 annotated genes were established for further bioinformatic analysis, performed with packages for the RStudio software. Raw data are deposited in the Gene Expression Omnibus repository and are accessible through GEO accession number GSE249434.

## Data analysis

### Samples and gene expression characterisation

**Fig. 1:**
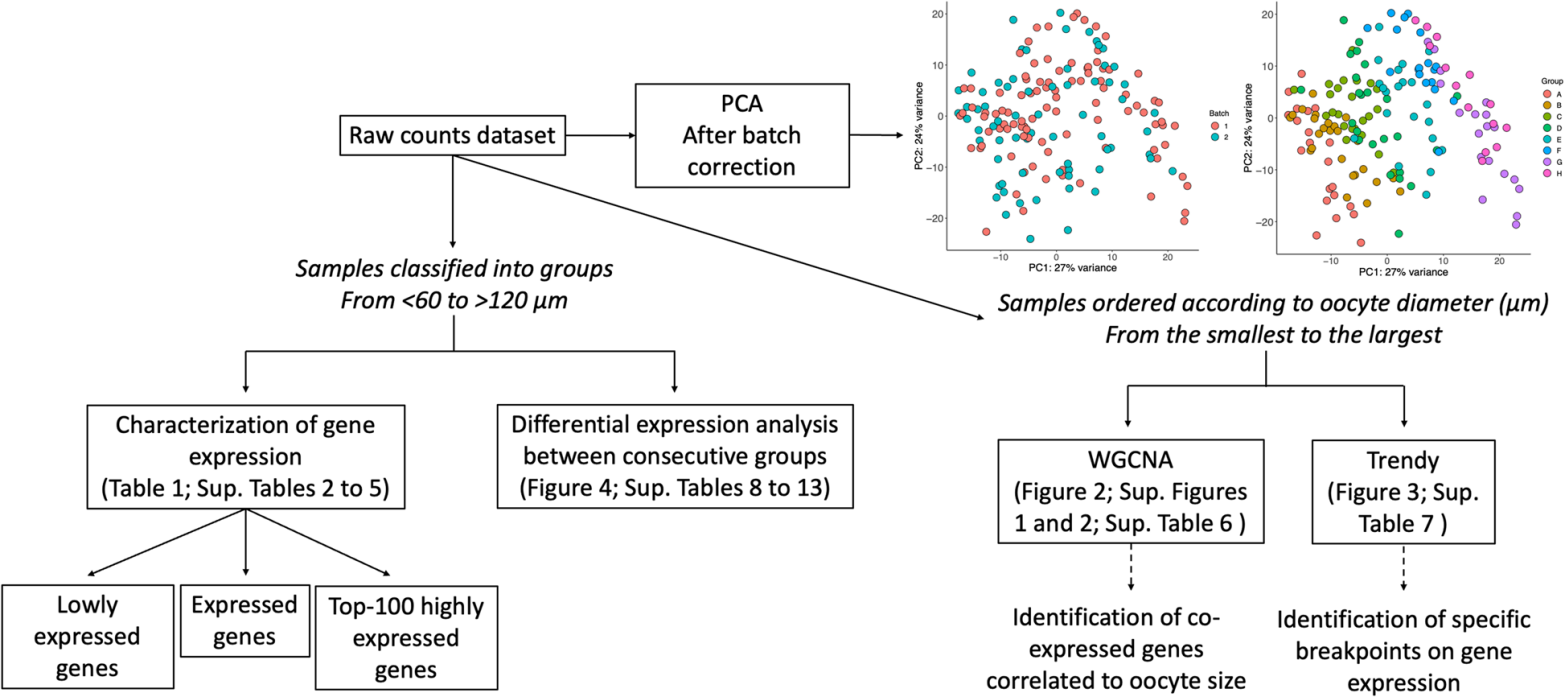
Data analysis pipeline. The scheme represents the workflow used to analyse samples of growing bovine oocytes differentiating when the oocytes were segregated in groups of 10 μm diameter from < 60 to > 120 μm or in a continuous order from the smallest to the largest. PCA: Principal Component Analysis plot highlighting batch and groups after batch correction. Oocyte diameter: **A** (< 60 μm), **B** (60–69 μm), **C** (70–79 μm), **D** (80–89 μm), **E** (90–99 μm), **F** (100–109 μm), **G** (110–119 μm), and **H** (> 120 μm)

A summary of the data analysis is represented in Fig. 1. Sample distribution was assessed by principal component analysis (PCA), which showed a gradual segregation of oocytes from the smallest to the largest size. Samples from different replicates clustered separately, indicating a batch effect (61% of variance) that was corrected using ComBat-seq function [33] (Fig. 1).

Initially, global gene expression characterization among groups according to oocyte size (< 60, 60–69, 70–79, 80–89, 90–99, 100–109, 110–119, and > 120 μm in diameter) was performed to identify the number of low abundant genes (zero count in half of the samples of each group), and consequently, expressed genes per group. In addition, the Top-100 genes were determined using counts per million to highlight the most abundantly expressed genes in each group.

### Statistical analyses

Three different statistical methods were chosen to best characterize key genes and pathways during bovine oocyte growth. First, to generate a broad view of gene expression and to identify groups of genes that change in expression according to oocyte size, weighted gene co-expression network analysis (WGCNA) was performed [34, 35]. Filtering was used to exclude genes below a K threshold determined by minimum count and sample library size. The top 50% most variable genes were subsequently divided into seven modules of co-expressed genes. The signed network construction and module detection were performed through the automatic method. Each module was given a first principal component called eigengene, which can be considered a representative of the gene expression profile in a module. Gene significance (GS) was calculated to determine the correlation of gene expression profiles (module eigengene) with an external trait *y*, oocyte size, and module membership (MM) was determined as the correlation between gene expression and module eigengene of a given module. The co-expression similarity was raised to a soft thresholding power (β) of 7 to calculate adjacency. The resulting co-expression modules were correlated with oocyte diameter to identify positive or negative correlation [34].

Second, the Trendy package [36] was applied to identify individual genes for which expression oscillated at a particular oocyte diameter. This method uses segmented regression models to identify patterns of gene expression in ordered conditions, such as oocyte size. The samples were ranked from the smallest to the largest size, and gene expression was classified as “up”, “down” or “no change” for each sample (adjusted *R*^*2*^ ≥ 0.5) when compared to the previous one.

Third, differential expression analysis was performed between successive oocyte diameter groups according to their size using the EdgeR package [37]. Raw p-values were adjusted using the Benjamini & Hochberg procedure. The DEGs were defined by those with a Fold Discovery Rate (FDR) < 0.05. The up and downregulated DEGs for each comparison were determined, and gene ontology (GO) analysis was performed with ShinyGO 0.77 using the protein-coding genome as background [38] to identify enriched pathways (FDR < 0.05).

## Results

### Global gene expression dynamics and transcriptional profile during bovine oocyte growth

For initial analysis of global gene expression during oocyte growth, the scRNA-seq datasets were binned according to oocyte size (< 60, 60–69, 70–79, 80–89, 90–99, 100–109, 110–119, and > 120 μm in diameter; Fig. 1). From the total of 16,979 genes detected after the exclusion of non-expressed genes (zero counts in all samples), the number of low expressed genes in each group (zero counts in at least half of the samples) was determined with an average of 5,463 genes (5,086 to 5,953) per group. Consequently, a mean of 11,516 genes (11,026 to 11,112) were considered expressed per group (Table 1) demonstrating limited variation and good turnover of expression during oocyte growth. A total of 10,320 genes were considered expressed in all groups. They were related to chromosome segregation, Golgi vesicle transport, regulation of translation, and establishment of organelle localization, among others (Supplementary Table S2), indicating that these pathways are active to a certain degree in all stages of oocyte growth. A small number of genes were identified as uniquely expressed in each group (Table 1, Supplementary Table S2), with the greatest number in oocytes < 60 μm in diameter (83 genes) and no genes uniquely expressed in 90–99 μm in diameter oocytes. Due to the small number, GO analysis did not find enriched pathways for the uniquely expressed genes.

**Table 1:** General analysis of gene expression in each experimental group

Group	Oocyte size (μm)	Follicle classification^a^	№ of samples	№ of lowly expressed genes^b^	№ of unique lowly expressed genes	№ of expressed genes	№ of unique expressed genes	№ of unique Top 100 expressed genes^c^
A	** < 60**	Secondary	21	5,542	173	11,437	83	4
B	**60–69**	Secondary	25	5,137	13	11,842	68	0
C	**70–79**	Transition	23	5,086	5	11,893	51	5
D	**80–89**	Early antral	24	5,145	3	11,834	48	3
E	**90–99**	Small antral	21	5,953	99	11,026	0	1
F	**100–109**	Medium antral	18	5,867	103	11,112	8	6
G	**110–119**	Large antral	19	5,464	35	11,515	38	9
H	** > 120**	Ovulatory	14	5,510	67	11,469	76	6
Common	-	-	165	4,365	-	10,320	-	-

^a^Follicle classification based on review by [8]

^b^Lowly expressed genes were identified as genes with zero counts in at least half of the samples for each group

^c^Top-100 genes are the highest expressed genes in each group according to mean counts

Regarding the low abundance genes, 4,365 were present in all groups and their associated pathways are summarized in Supplementary Table S3. They include extracellular matrix organization, potassium ion transmembrane transport, and adaptive immune response. A number of genes were uniquely lowly expressed in each group, the greatest number of which (173 genes) were identified in < 60 μm diameter oocytes (Table 1, Supplementary Table S3). Significantly enriched pathways were not assigned for any of the uniquely lowly expressed genes.

The Top-100 genes with the highest counts per million were ranked to detect the most abundant genes in each oocyte diameter group. Interestingly, while GO analysis revealed similar pathways populated across the groups, telomere maintenance and organization and histone H2B ubiquitination pathways were uniquely enriched in 60–69 μm and 110–119 μm diameter oocytes, respectively (Supplementary Table S4). A total of 48 Top-100 genes were common to all groups and a smaller number (1 to 9) were uniquely highly expressed in each group (summarized in Table 1; genes listed in Supplementary Table S5).

### Size-specific signature gene clusters identified in bovine oocytes

**Fig. 2:**
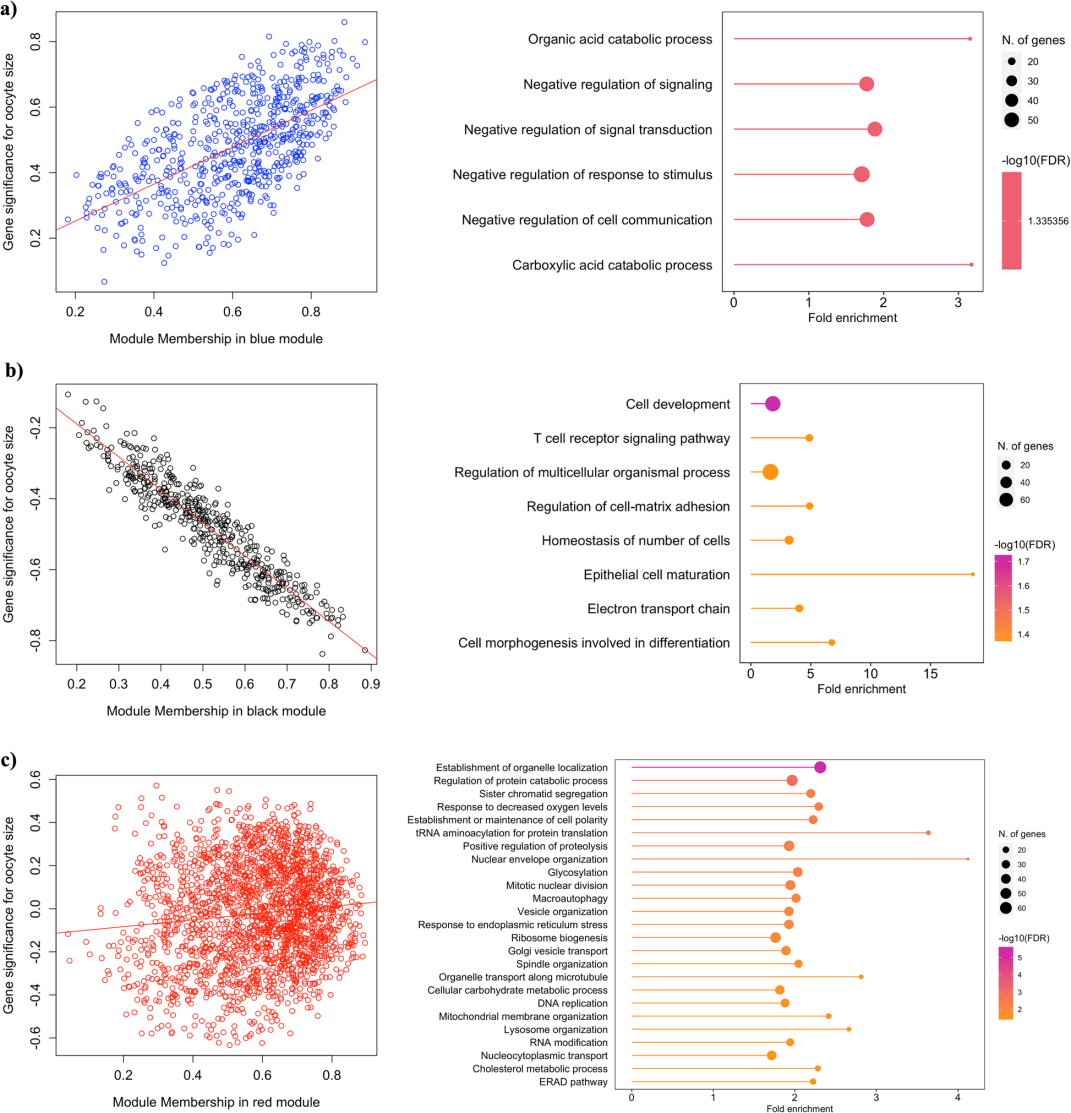
Modules of co-expressed genes correlated with oocyte size identified by WGCNA. Module Blue (**a**), Black (**b**), and Red (**c**) scatterplot between gene significance (correlation of gene expression profiles with an external trait *y*, oocyte size) and module membership (correlation between gene expression profile and module eigengene of a given module) and respectively enriched pathways from gene ontology analysis

Seven clusters of co-expressed genes correlated with oocyte size were identified by WGCNA (Supplementary Fig. S2), of which only two had a high correlation. The Blue module clustered genes that are positively correlated with oocyte size (*r* = 0.78; *p* < 0.01; 652 genes), indicating that their expression increases during oocyte growth. Further, GO analysis using ShinyGO 0.77 using the protein-coding genome as background (FDR < 0.05) revealed response to stimulus, cell communication, and negative regulation of signalling as the most enriched biological pathways in this gene cluster (Fig. 2a). In contrast, the expression of genes in the Black module (*r* = -0.94; *p* < 0.01; 472 genes) was negatively correlated with oocyte size and was populated by genes related to cell development, regulation of cell–matrix adhesion, and electron transport chain, among others (Fig. 2b). Interestingly, the highest number of genes were clustered in the Red module and were weakly correlated with oocyte size (*r* = -0.026; *p* = 0.7; 2,053 genes), indicating their stable expression throughout the growth phase. The main pathways in which these genes were enriched included the establishment of organelle localisation, protein catabolic process, tRNA aminoacylation for protein translation, response to decreased oxygen levels, nuclear envelope organization, and glycosylation, among others (Fig. 2c). A complete list of the identified genes in each module and their associated GO pathways as they relate to oocyte diameter, are provided in Supplementary Table S6 and Supplementary Fig. S3. Of particular note is the increased expression of genes involved in cell communication and decreased expression of the electron transport chain pathway components during bovine oocyte growth.

### Identification of stage-specific genes during oocyte growth

**Fig. 3:**
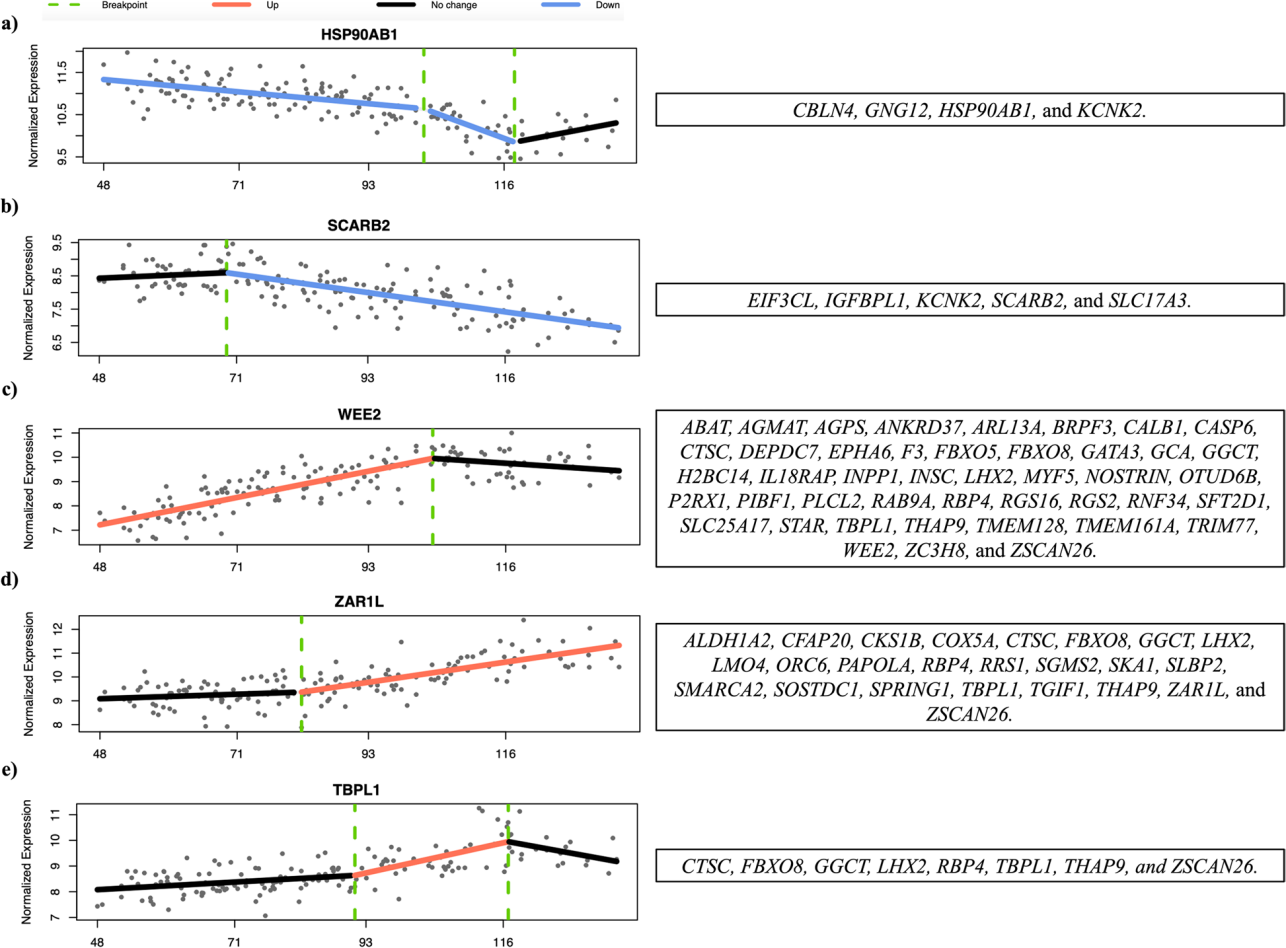
Genes selected by Trendy package that **a** decrease expression until a certain oocyte size and do not change after the breakpoint; **b** have a stable expression until a certain oocyte size and decrease after the breakpoint; **c** increase expression until a certain oocyte size and do not change after the breakpoint; **d** have a stable expression until a certain oocyte size and increase after the breakpoint; **e** increase in expression in the middle of the oocyte growth phase, having a stable expression before and after the breakpoints. The graphs show the normalised expression by Variance Stabilizing Transformation (DESeq2) across all samples ordered by the smallest to the biggest oocyte diameter (μm)

A smaller cohort of genes (162) was identified by Trendy analysis as significantly changing in expression at a specific stage of oocyte growth. Many of these were also classified by WGCNA analysis as positively (58) or negatively (25) correlated with oocyte diameter. In addition, 5 additional patterns of expression were determined, which are represented in Fig. 3. For example, the expression of the genes *SLBP2* and *ZARL1* was initially stable and then increased after oocytes reached diameters of 70 and 80 μm, respectively. In contrast, the expression of *RGS2* and *WEE2* increased until oocytes attained a diameter of 100 μm, becoming stable after that point. All significantly expressed genes and respective breakpoints are listed in Supplementary Table S7. The identification of stage-specific expressed genes highlights the dynamic nature of their functional contribution to oocyte growth and acquisition of developmental competence.

### Dramatic change in oocyte transcriptome at late stages of oocyte growth

**Fig. 4:**
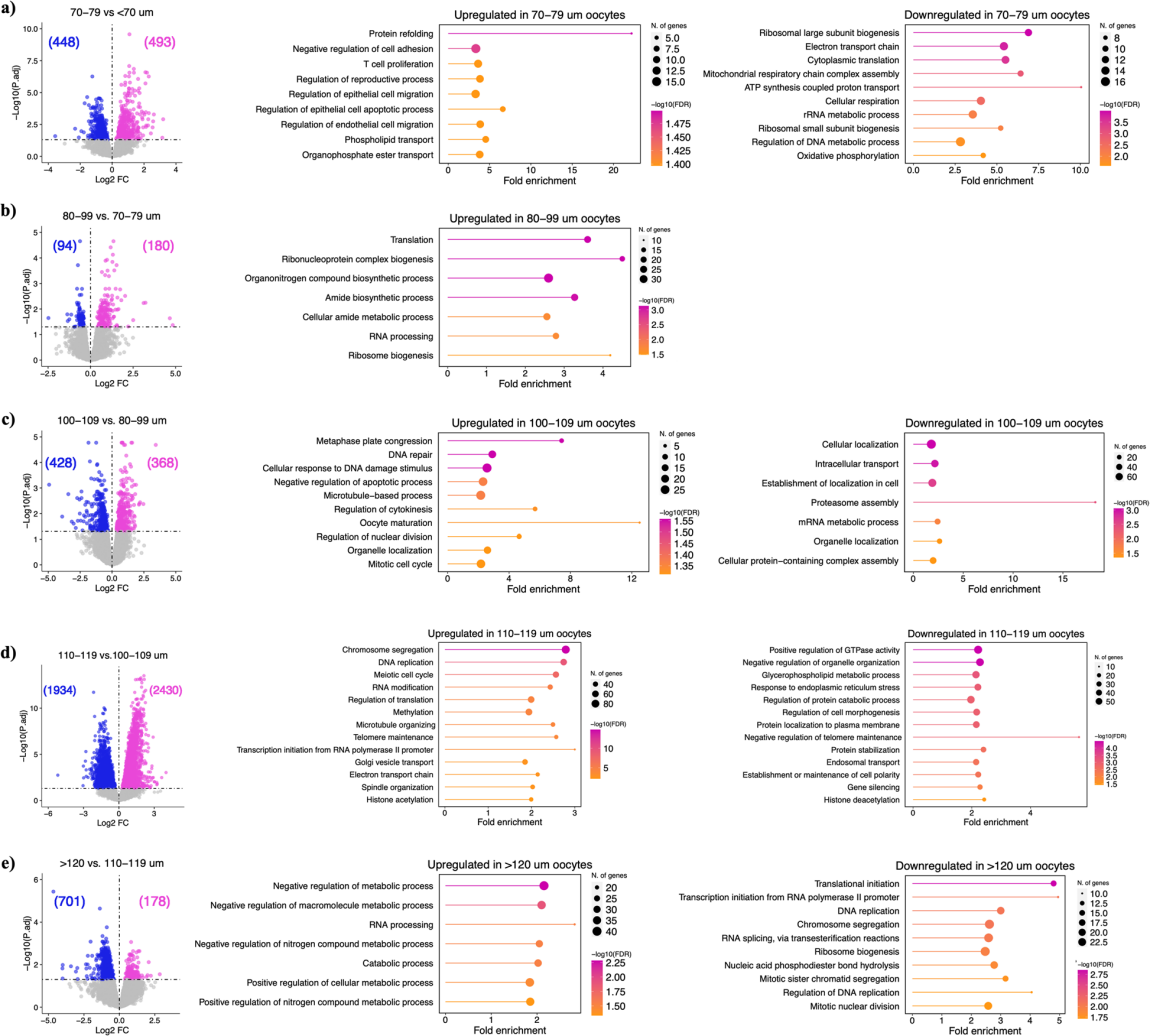
Differential expression analysis. Volcano plot of the DEGs and respective up and downregulated pathways from GO analysis between the groups (**a**) < 70 and 70–79 μm oocytes; (**b**) 70–79 and 80–99 μm oocytes; (**c**) 80–99 and 100–109 μm oocytes; (**d**) 100–109 and 110–119 μm oocytes; (**e**) 110–119 and > 120 μm oocytes. Upregulated genes are represented in pink and downregulated genes are represented in blue

To identify DEGs during oocyte growth, we initially compared the eight oocyte diameter groups in sequential order of ascending size. Because only 35 genes were differentially expressed between 60 and 60–69 μm diameter oocytes and two genes between 80–89 and 90–99 μm, these groups were combined, resulting in the final six oocyte diameter groups of < 70, 70–79, 80–99, 100–109, 110–119 and > 120 μm for this analysis. The differential expression analysis revealed 941 DEGs for the comparison of < 70 and 70–79 μm, among which the upregulation of maternal-effect genes (*BMP15, GDF9, NLRP5, PADI6, ZP2, ZP3, ZP4*) in 70–79 μm oocytes is particularly noteworthy. GO analysis revealed protein refolding, negative regulation of cell adhesion, and phospholipid transport as upregulated pathways, and ribosome biogenesis and mitochondrial respiratory chain as downregulated in 70–79 μm in diameter oocytes (Fig. 4a; Supplementary Table S8). A total of 274 DEGs were detected in the comparison of 70–79 and 80–99 μm diameter oocytes, including the upregulation of maternal-effect genes *DPPA3*, *FABP3*, and *OOSP2* and the pathways ribonucleoprotein complex, ribosome biogenesis, translation, and RNA processing in 80–99 μm oocytes (Fig. 4b; Supplementary Table S9). The list of downregulated genes (94) included *IDH3B*, *KDM5A*, *SOD2*, *STT3A*, and *ZP4*, however, GO terms were not identified. The comparison between 80–99 and 100–109 μm oocyte diameter groups showed 796 DEGs. GO analysis revealed oocyte maturation and development, mitotic metaphase plate congression, microtubule-based process, and progesterone-mediated oocyte maturation as significantly upregulated pathways, in addition to organelle localization, mRNA metabolic process, and intracellular transport as downregulated pathways in 100–109 μm oocytes (Fig. 4c; Supplementary Table S10). The highest number of DEGs (4,364) was identified in the comparison of gene expression in 100–109 versus 110–119 μm diameter groups. Enriched upregulated pathways included chromosome and sister chromatid segregation, DNA repair, and meiotic cell cycle, while negative regulation of organelle organization, positive regulation of GTPase activity, and negative regulation of telomere maintenance were enriched downregulated pathways in 110–119 μm oocytes (Fig. 4d; Supplementary Table S11). The final analysis, comparing 110–119 and > 120 μm diameter oocytes, identified 178 upregulated DEGs, associated with negative regulation of metabolic pathways, cellular catabolic processes, and RNA processing in > 120 μm oocytes. A higher number of DEGs (701) were downregulated and they were assigned to the regulation of DNA-directed DNA polymerase activity, translational initiation, protein localization to endoplasmic reticulum, sister chromatid segregation, and DNA replication, among others (Fig. 4e; Supplementary Table S12). Following the differential expression analysis, 5,520 genes were identified which did not change significantly across any of the comparisons described above. These genes may be described as having a stable expression throughout bovine oocyte growth. They are involved in protein modification and transport, mitotic spindle organization, methylation, histone modification, and mRNA splicing, among others (Supplementary Table S13). Many of these genes (~ 1,000) were also present in the WGCNA modules (Green and Red) with a weak correlation between expression and oocyte size, supporting a stable expression profile across the bovine oocyte growth phase. The differential expression analysis revealed the progressive and sometimes dramatic changes in gene expression from one oocyte size group to the other, indicating the diversity and necessity of specific genes according to the stage of oocyte development.

## Discussion

Employing scRNA-seq, the present study provides a detailed characterisation of the bovine oocyte transcriptome from the onset of transcription in the secondary follicle to the completion of the growth phase and cessation of transcription in the oocyte of the mid-antral follicle. Here we present novel knowledge identifying a panel of core genes whose stable expression is likely essential to maintain homeostasis and provide the optimal environment for stage-specific processes to take place and reveal the genes and pathways that are differentially regulated at specific stages of oocyte growth.

The interpretation of the scRNA-seq data was carried out with the knowledge that the artificial selection of long poly(A) tails mRNA by the RNA extraction technique may not reflect the full biological picture. While the bovine model permits functional analyses during maturation, fertilization and early cleavage stages in vitro, the lack of a robust in vitro oocyte growth culture system restricts our ability to study specific gene functions during oocyte growth, at present.

### Mammalian oocyte growth-associated transcriptome changes: shared and species-specific features

When compared to datasets from similar studies, our data shows greater similarities with data from human growing oocytes than from mouse. We identified 21,218 transcripts in bovine oocytes compared to 20,100 in humans [39] and 21,402 – 32,775 in mouse [14]. From the 21,218 genes, 11,112—11,893 genes met our stringency criteria for inclusion as an expressed gene in our dataset, within which the expression of 5,520 did not change significantly across the bovine oocyte growth phase. In stark contrast to data from mouse, where fewer than 4% of genes were differentially expressed during the oocyte growth phase [14], we identified ~ 5000 DEGs across consecutive stages of oocyte development in our study. Moreover, this scale of dynamic gene expression is comparable to human oocyte growth, where 2,326 DEGs were detected in primary follicle oocytes [39], highlighting the relevance of the bovine oocyte model to human fertility research.

### Changes in energy production during oocyte growth: expression of transcripts related to oxidative phosphorylation is negatively correlated with oocyte size

To identify key genes and pathways specific to each stage of bovine oocyte development, several methods of data analysis were employed, allowing us to scrutinize genes whose expression changes according to the oocyte diameter. A cohort of genes, mainly associated with energy production through the electron transport chain, was identified by WGCNA as negatively correlated with oocyte size. Generally, pyruvate consumption is higher in oocytes from primordial through secondary follicles (reviewed by [40]). At the time of antrum formation, there is an increase in glycolytic activity, especially when oxygen availability is limited in the late preantral to early antral stage and at the early preovulatory stage [41, 42]. The expression pattern of genes involved in oxidative phosphorylation agrees with these studies, since it was highest in early (< 70 μm) and late (110–119 μm) growth-stage oocytes, suggesting a balance between glycolysis and oxidative phosphorylation in the mid-phase of oocyte growth.

### Genes positively correlated to oocyte size

The expression of a cohort of genes (103) was highly positively correlated with oocyte diameter (> 0.7). The cohort includes the maternal factor *DPPA3*, the estrogen receptor *ESRRG*, the oocyte-specific histone RNA binding protein *SLBP2*, and the oocyte meiosis inhibitor *WEE2*. A similar correlation analysis was undertaken in mouse oocytes [43]. Comparing the two datasets, we determined the expression pattern of *DCLRE1A, OOSP2, OTUD6B, RGS2, STK35,* and *ZAR1L* to be conserved between the two species. Furthermore, WGCNA was also used to analyze human oocyte samples from primordial to pre-ovulatory follicles with the identification of a module of co-expressed genes that were highly correlated (0.78) to oocytes from antral follicles [44]. Several of which, including *ACTL8, BCAR4, GDF9, KHDC3L, NLRP2, ZARL1,* and *WEE2,* were also present in our list of genes positively correlated to oocyte size, indicating their importance to the development of oocytes in antral follicles in multiple species.

The genes that increased in expression across the bovine oocyte growth phase were primarily associated with mechanisms that enable the oocyte to successfully complete maturation and fertilization, particularly genes related to chromosome segregation, meiotic nuclear division, centromere complex assembly, and cytoskeleton organization. These pathways were preferentially upregulated in oocytes > 100 μm in diameter. Thus, our data highlights the importance of the last phase of transcription, as the bovine oocyte reaches the fully-grown stage, for accumulating transcripts required for the resumption of meiosis and completion of maturation.

Well-orchestrated changes occur in the oocyte nucleus before maturation. Chromatin condensation and cessation of transcription are hallmarks of the end of the oocyte growth phase [45]. Many of the DEGs were downregulated in the largest oocyte diameter group (> 120 μm), likely related to the reduction in transcription activity at this stage. Conversely, genes associated with chromatin condensation and suppression of transcription were upregulated. For example, *DPPA3*, also known as *STELLA*, is essential for chromatin condensation as *Dppa3*-null mice oocytes failed to progress from a non-surrounded nucleus (NSN) to a surrounded nucleus (SN) and to repress transcription [46]. This function may be conserved in bovine since the observed increase in *DPPA3* parallels the first signs of decreased mRNA transcription and nucleolus deactivation in oocytes from 100–110 μm in diameter [11, 45, 47]. Histone modification also plays an important role in the cessation of transcription and increased H3K4me3 and H3K9me3 are related to inactivation of the oocyte transcriptome [48]. Here we report an incremental increase in histone methyltransferases and a reduction in histone deacetylases and demethylases, in addition to increased expression of *H3-3B* and *SLBP2*, which protect histones from early translation [49], during the final stages of oocyte growth (110–119 μm diameter group).

One of the main objectives of transcription during oocyte growth is the accumulation and storage of mRNA to support oocyte maturation and embryo development until the embryonic genome is activated [45, 50]. Indeed, *ZAR1L* expression increased throughout bovine oocyte growth and was one of the unique Top-100 expressed genes in oocytes > 120 μm in diameter. The proteins ZAR1 and ZAR2 (also known as ZAR1L) were previously identified as RNA-binding proteins participating in mRNA storage and oocyte maturation [51, 52]. Thus, the increase in *ZAR1L* expression likely indicates its role in mRNA storage towards the end of the bovine oocyte growth phase. We also identified candidate genes that might be preferentially transcribed for storage and later translation, since their main function is related to oocyte maturation, including *WEE2, OOSP2,* and *RSG2*. *WEE2* is known to prevent premature resumption of oocyte meiosis, but it also plays a role in metaphase II progression during fertilization [53]. The oocyte-specific protein *OOSP2* is required during maturation [54] together with *RGS2*, which inhibits early Ca^2+^ release [55] and helps with chromosome segregation through association with β-tubulin [56].

### The expression of maternal-effect genes

In addition to the identification of genes associated with oocyte diameter discussed above, the expression pattern of oocyte-specific and maternal-effect genes is noteworthy. Expression of *BMP15*, *NLRP2*, *NLRP5*, *PADI6*, and *TLE6* was highest in the mid-phase of oocyte growth (from 70 to 110 μm in diameter). The oocyte-secreted factor *BMP15* is critical for follicle development and cell proliferation (reviewed by [57]), with no development beyond the primary follicle stage in sheep with spontaneous homozygous mutations in the *BMP15* [58]. Thus, the observed increase in *BMP15* from the late secondary to the mid-antral follicle aligns with its role in supporting follicle growth. The maternal-effect genes *NLRP2*, *NLRP5*, *PADI6*, and *TLE6* encode members of the subcortical maternal complex (SCMC) and/or cytoplasmic lattices multiprotein complexes, essential for early embryonic development (reviewed by [59, 60]). These complexes are involved in organelle distribution [61], mRNA storage [62], spindle localisation [63], and epigenetic reprogramming [64–66]. The increase in their expression in mid-growth phase oocytes likely reflects the critical importance of this stage of development for oocyte acquisition of competence, indicating a possible window for storage of proteins involved in early embryo development.

## Conclusions

In conclusion, the results presented here bring novel information contributing to a better understanding of the events occurring during bovine oocyte growth. We revealed the emphasis on the expression of genes associated with cytoplasmic activity and function during the early stages of the oocyte growth phase, upregulation of oocyte-specific and maternal-effect genes during the mid-stages, and a shift to transcripts associated with nuclear activity, particularly preparation for the resumption of meiotic maturation at the end of the growth phase. This is the first study to characterise the transcriptome of bovine oocyte growth at single-cell resolution. It provides a unique resource for researchers and practitioners working to optimise oocyte developmental competence by targeting bovine oocyte growth in vivo and in vitro.

### Supplementary Information

**Additional file 1: Supplementary Figure S1. **High sensitivity DNA Assay. Amplified cDNA was evaluated by a High Sensitivity DNA Assay chip (Agilent Technologies). Individual samples were pooled and diluted in different concentrations (triplicate). The results were used to choose the best dilution for final library preparation and sequencing.

**Additional file 2: Supplementary Figure S2. **Network analysis of the transcriptome across oocyte growth. **a)** Hierarchical cluster tree showing co-expression modules identified using WGCNA. Modules correspond to branches and are labelled by colours. **b)** Heatmap showing the correlation (r) and significance (p-value) of different modules associated with oocyte size. The colour scale on the right (green to red) corresponds to the correlation value (-1 to 1).

**Additional file 3: Supplementary Figure S3. **Modules of co-expressed genes moderately correlated with oocyte size. Module Turquoise (1,356 genes) **(a)**, Yellow (529 genes) **(b)**, and Green (168 genes) **(c)** correlation graph between gene expression and oocyte size and respectively enriched pathways from gene ontology analysis.

**Additional file 4: Supplementary Tables. Table S1.** Sample quality control analysis performed in Rstudio by selecting samples with more than 100,000 reads and 2,500 expressed genes (genes with more than 1 count). In addition, filtering for contamination with mitochondrial DNA was also performed. Additional information is from MuitQC Report. The table on the right summarizes the information by experimental groups. **Table S2.** Common and unique expressed genes (more than 1 count) in each oocyte size group. Enriched pathways from gene ontology analysis are shown in the panel on the right. **Table S3.** Common and unique lowly expressed genes (zero count in at least half of the samples for each group) in each oocyte size group. Enriched pathways from gene ontology analysis are shown in the panel on the right. **Table S4.** Top-100 highly expressed genes on each oocyte size group. Enriched pathways from gene ontology analysis are shown in the panels on the right. **Table S5.** Common and unique Top-100 highly expressed genes in each oocyte size group. Enriched pathways from gene ontology analysis are shown in the panel on the right. **Table S6:** Summary table of WGCNA output. GS/p.GS: gene significance for oocyte size and related p-value. The higher the absolute value, the more biologically relevant to oocyte size. MM/p.MM: Module membership and related p value. Correlation value of each gene related to gene expression profile in a module. **Table S7.** Trendy analysis output table to identify changes in gene expression of specific genes. Segment slope measures the rate of change of the data within that segment. The segment trend defines how the expression of each gene changes inside that segment. 1 = increase; -1 = decrease; 0/NA = no change. The p-value will indicate if the change is significant (<0.05). Breakpoints indicate the oocyte diameter at which gene expression is changing. Only the top dynamic genes are shown, defined as those whose optimal model has a high adjusted R2 (>0.5). **Table S8.** Differentially expressed genes between <70 and 70-79 um in diameter oocytes (up and downregulated). Significant genes were determined by FDR<0.05. Enriched pathways from gene ontology analysis are presented in the panels on the right. **Table S9.** Differentially expressed genes between 70-79 and 80-99 um in diameter oocytes (up and downregulated). Significant genes were determined by FDR<0.05. Enriched pathways from gene ontology analysis are presented in the panel on the right. **Table S10.** Differentially expressed genes between 80-89 and 100-109 um in diameter oocytes (up and downregulated). Significant genes were determined by FDR<0.05. Enriched pathways from gene ontology analysis are presented in the panels on the right. **Table S11.** Differencial expressed genes between 100-109 and 110-119 um in diameter oocytes (up and downregulated). Significant genes were determined by FDR<0.05. The panels on the right are showing all the enriched pathways from gene ontology analysis. **Table S12.** Differential expressed genes between 110-119 and >120 um in diameter oocytes (up and downregulated). Significant genes were determined by FDR<0.05. Enriched pathways from gene ontology analysis are presented in the panel on the right. **Table S13.** Genes with stable expression across the bovine oocyte growth phase. Respective enriched pathways are listed in the table on the right.

## Data Availability

The datasets generated during the current study are available in the GEO repository, GSE249434.

## Abbreviations

ACTL8: Actin Like 8
BCAR4: Breast Cancer Anti-Estrogen Resistance 4
BMP15: Bone Morphogenetic Protein 15
BSA: Bovine Serum Albumin
CA2 +: Calcium ions
DCLRE1A: DNA Cross-Link Repair 1A
DEGs: Differently Expressed Genes
DPPA3/STELLA: Developmental pluripotency-associated 3
ESRRG: Estrogen Related Receptor Gamma
FABP3: Fatty Acid Binding Protein 3
FDR: Fold Discovery Rate
GDF9: Growth Differentiation Factor 9
GO: Gene Ontology
GV: Germinal Vesicle
H3-3B: H3.3 Histone B
H3K4me3: Histone H3 lysine 4 tri-methylation
H3K9me3: Histone H3 lysine 9 tri-methylation
IDH3B: Isocitrate Dehydrogenase (NAD( +)) 3 Non-Catalytic Subunit Beta
KDM5A: Lysine Demethylase 5A
KHDC3L: KH Domain Containing 3 Like, Subcortical Maternal Complex Member
MII: Metaphase II
mRNA: Messenger RNA
NLRP2: NLR Family Pyrin Domain Containing 2
NLRP5: NLR Family Pyrin Domain Containing 5
NSN: Non-Surrounded Nucleus
OOSP2: Oocyte Secreted Protein 2
OTUD6B: OTU Deubiquitinase 6B
PADI6: Peptidyl Arginine Deiminase 6
PBS: Phosphate Basic Solution
PCA: Principal Component Analysis
RGS2: Regulator of G-Protein Signalling 2
RNA-seq: RNA sequencing
SCMC: Subcortical maternal complex
scRNA-seq: Single-cell RNA sequencing
SLBP2: Oocyte-Specific Histone RNA Stem-Loop-Binding Protein 2-Like
SN: Surrounded Nucleus
SOD2: Superoxide Dismutase 2
STK35: Serine/Threonine Kinase 35
STT3A: STT3 Oligosaccharyltransferase Complex Catalytic Subunit A
TCM: Tissue Culture Medium
TLE6: TLE Family Member 6
tRNA: Transfer RNA
WEE2: WEE2 Oocyte Meiosis Inhibiting Kinase
WGCNA: Weighted Gene Co-expression Network Analysis
ZAR1: Zygote Arrest 1
ZAR2: Zygote Arrest 2
ZARL1: Zygote Arrest 1 Like
ZP2: Zona Pellucida Glycoprotein 2
ZP3: Zona Pellucida Glycoprotein 3
ZP4: Zona Pellucida Glycoprotein 4
