# Supplementary material for: Single-cell profiling reveals transcriptome dynamics during bovine oocyte growth
Source: BMC Genomics. 2024 Apr 6;25:335. doi: 10.1186/s12864-024-10234-0 (PMC10998374; doi:10.1186/s12864-024-10234-0)
Supplement: Supplementary file 1 — Additional file 1: Supplementary Figure S1. High sensitivity DNA Assay. Amplified cDNA was evaluated by a High Sensitivity DNA Assay chip (Agilent Technologies). Individual samples were pooled and diluted in different concentrations (triplicate). The results were used to choose the best dilution for final library preparation and sequencing. [file 12864_2024_10234_MOESM1_ESM.pdf]

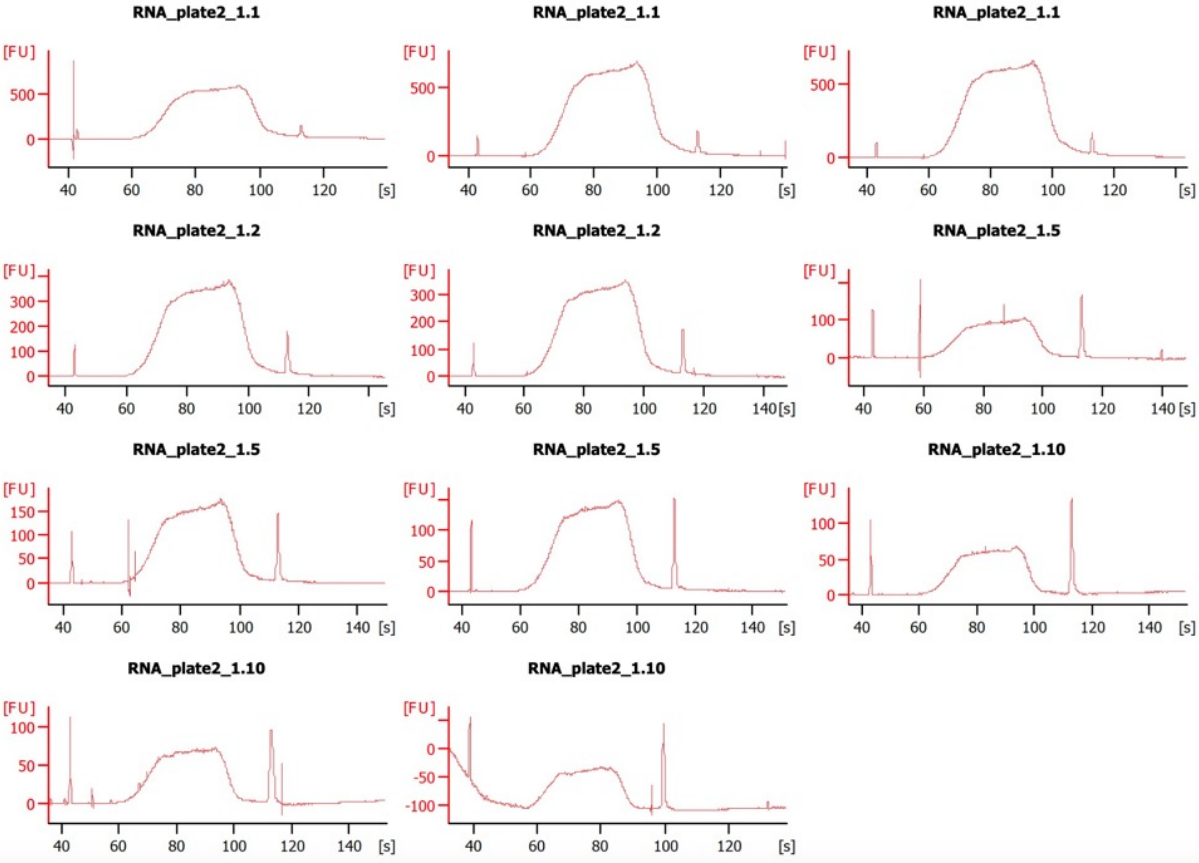

RNA_plate2_1.1
[FU]
500
0
40 60 80 100 120 [s]
RNA_plate2_1.1
[FU]
500
0
40 60 80 100 120 [s]
RNA_plate2_1.1
[FU]
500
0
40 60 80 100 120 [s]
RNA_plate2_1.2
[FU]
300 200 100 0
40 60 80 100 120 [s]
RNA_plate2_1.2
[FU]
300 200 100 0
40 60 80 100 120 140 [s]
RNA_plate2_1.5
[FU]
100 0
40 60 80 100 120 140 [s]
RNA_plate2_1.5
[FU]
150 100 50 0
40 60 80 100 120 140 [s]
RNA_plate2_1.5
[FU]
100 50 0
40 60 80 100 120 140 [s]
RNA_plate2_1.10
[FU]
100 50 0
40 60 80 100 120 140 [s]
RNA_plate2_1.10
[FU]
100 50 0
40 60 80 100 120 140 [s]
RNA_plate2_1.10
[FU]
0 -50 -100
40 60 80 100 120 [s]
