# Supplementary figures and images for: Single-cell profiling reveals transcriptome dynamics during bovine oocyte growth
Source: BMC Genomics. 2024 Apr 6;25:335. doi: 10.1186/s12864-024-10234-0 (PMC10998374; doi:10.1186/s12864-024-10234-0)

a)

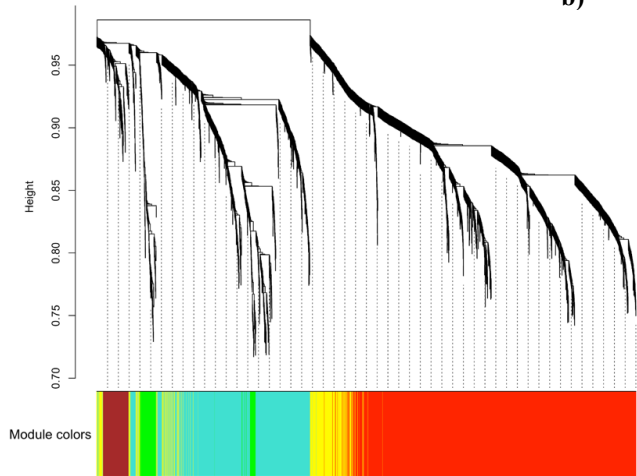

b)

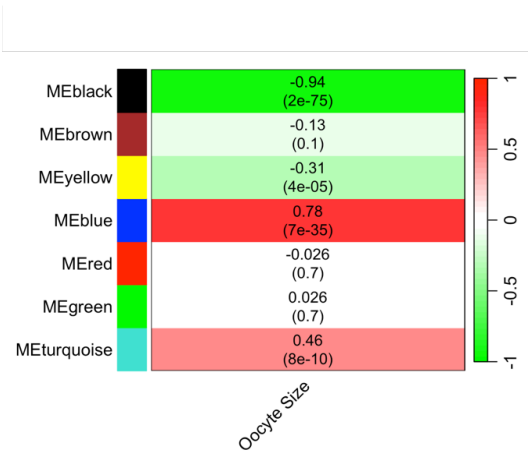

Supplement: Supplementary file 2 — Additional file 2: Supplementary Figure S2. Network analysis of the transcriptome across oocyte growth. a) Hierarchical cluster tree showing co-expression modules identified using WGCNA. Modules correspond to branches and are labelled by colours. b) Heatmap showing the correlation (r) and significance (p-value) of different modules associated with oocyte size. The colour scale on the right (green to red) corresponds to the correlation value (-1 to 1). [file 12864_2024_10234_MOESM2_ESM.pdf]

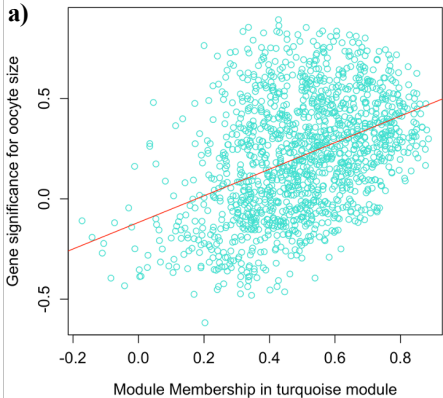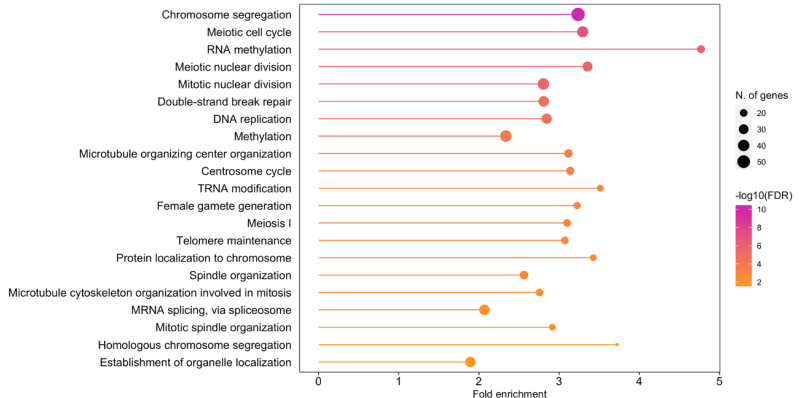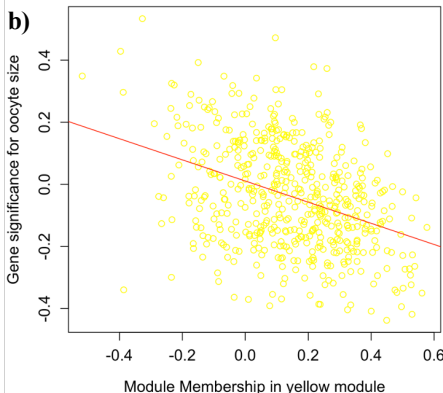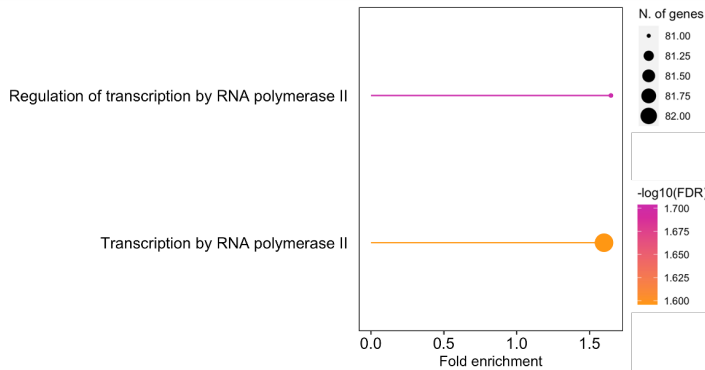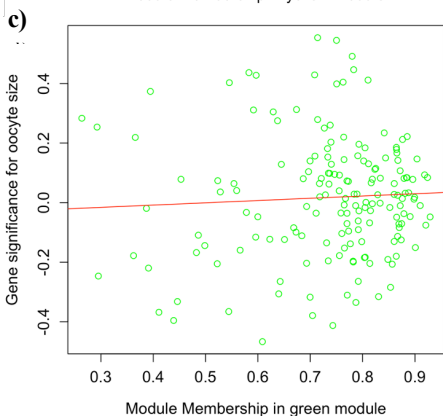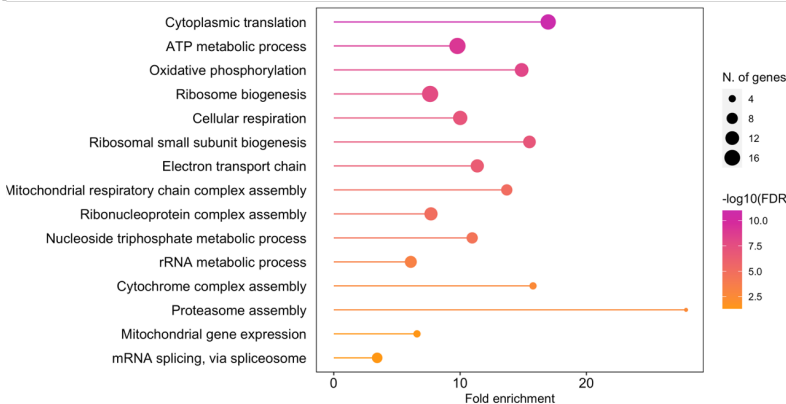

Supplement: Supplementary file 3 — Additional file 3: Supplementary Figure S3. Modules of co-expressed genes moderately correlated with oocyte size. Module Turquoise (1,356 genes) (a), Yellow (529 genes) (b), and Green (168 genes) (c) correlation graph between gene expression and oocyte size and respectively enriched pathways from gene ontology analysis. [file 12864_2024_10234_MOESM3_ESM.pdf]
